# T3SEdb: data warehousing of virulence effectors secreted by the bacterial Type III Secretion System

**DOI:** 10.1186/1471-2105-11-S7-S4

**Published:** 2010-10-15

**Authors:** Daniel Ming Ming Tay, Kunde Ramamoorthy Govindarajan, Asif M Khan, Terenze Yao Rui Ong, Hanif M Samad, Wei Wei Soh, Minyan Tong, Fan Zhang, Tin Wee Tan

**Affiliations:** 1 Department of Biochemistry, Yong Loo Lin School of Medicine, National University of Singapore, Singapore 117597

## Abstract

**Background:**

Effectors of Type III Secretion System (T3SS) play a pivotal role in establishing and maintaining pathogenicity in the host and therefore the identification of these effectors is important in understanding virulence. However, the effectors display high level of sequence diversity, therefore making the identification a difficult process. There is a need to collate and annotate existing effector sequences in public databases to enable systematic analyses of these sequences for development of models for screening and selection of putative novel effectors from bacterial genomes that can be validated by a smaller number of key experiments.

**Results:**

Herein, we present T3SEdb http://effectors.bic.nus.edu.sg/T3SEdb, a specialized database of annotated T3SS effector (T3SE) sequences containing 1089 records from 46 bacterial species compiled from the literature and public protein databases. Procedures have been defined for i) comprehensive annotation of experimental status of effectors, ii) submission and curation review of records by users of the database, and iii) the regular update of T3SEdb existing and new records. Keyword fielded and sequence searches (BLAST, regular expression) are supported for both experimentally verified and hypothetical T3SEs. More than 171 clusters of T3SEs were detected based on sequence identity comparisons (intra-cluster difference up to ~60%). Owing to this high level of sequence diversity of T3SEs, the T3SEdb provides a large number of experimentally known effector sequences with wide species representation for creation of effector predictors. We created a reliable effector prediction tool, integrated into the database, to demonstrate the application of the database for such endeavours.

**Conclusions:**

T3SEdb is the first specialised database reported for T3SS effectors, enriched with manual annotations that facilitated systematic construction of a reliable prediction model for identification of novel effectors. The T3SEdb represents a platform for inclusion of additional annotations of metadata for future developments of sophisticated effector prediction models for screening and selection of putative novel effectors from bacterial genomes/proteomes that can be validated by a small number of key experiments.

## Background

The Type III Secretion System (T3SS) is an essential mechanism for host-pathogen interaction during the infection process and is found in many gram-negative bacteria pathogens and eukaryotic cell symbionts [1]. Examples include *Yersinia *spp., *Salmonella *spp., *Burkholderia*, *Pseudomonas *and *Chlamydia *[2]. The T3SS machinery is a highly conserved multi-protein apparatus that mediates the delivery of bacterial effector proteins into the host cell [3]. T3SS effector (T3SE) proteins act as virulence factors within the host and are able to alter and manipulate vital host cell functions, such as signal transduction [4] and innate immune response [5].

Due to the key role of T3SE proteins in the establishment and maintenance of bacterial pathogenicity, there is considerable research interest in the identification of T3SS effectors. However T3SEs display high level of sequence diversity, due largely to horizontal gene transfer among evolutionarily distant species and subsequent bacterial adaptation to different host cell environments [6]. To date, while quite a number of T3SEs have been identified by both *in vitro *and *in silico *methods, the rising number of effector sequences being discovered each year suggests that this represents only a small proportion of all effectors, with many more yet to be discovered.

There is a need to collate and annotate these known effector sequences to enable systematic analyses of these sequences for development of models for screening and selection of putative novel effectors from bacterial genomes/proteomes that can be validated by a small number of key experiments. There is no publicly available specialized database of T3SEs, although databases exist for the T3SS machinery, such as the Database of Type 3 Secretion System (DTTSS) [7]. Herein, we present T3SEdb, a specialized database of annotated T3SS effectors, Web-accessible at http://effectors.bic.nus.edu.sg/T3SEdb. By using the reported annotated repertoire of effectors in the database, we have built a reliable T3SS effector prediction model that may be useful for predicting T3SS effectors expressed in a broad spectrum of bacterial species.

## Results and discussion

### T3SEdb data

T3SEdb contains 1089 cross-referenced and manually annotated effector records (as of April 2010), of which 504 are experimentally verified (E), 572 hypothetical (H) and 13 unknown (U), originating from a total of 46 bacterial species. Nine bacterial species (*Escherichia coli, Salmonella enteric, Citrobacter rodentium, Pseudomonas syringae, Yersinia pestis, Chlamydia trachomatis, Shigella flexneri, Yersinia enterocolitica *and *Burkholderia pseudomallei*) had more than 10, both experimentally verified and hypothetical effector sequences, with *Escherichia coli *having the most reported.

T3SEdb records are built on effector sequence records retrieved using various keywords from the NCBI Entrez Protein database. The original records were manually checked to remove irrelevant records and the retained records were processed to customise the data for the specialised T3SEdb by keeping only fields of interest (list of fields is provided at: http://effectors.bic.nus.edu.sg/T3SEdb/fielddescription.php). A T3SEdb record is assigned a unique five character identifier, which consists of a single letter "T" followed by four numeric digits. The experimental status of each record (either E, H or U) was defined following the comprehensive annotation procedure that we defined http://effectors.bic.nus.edu.sg/T3SEdb/annotationpolicy.php, which involved manually scanning through the literature via PubMed [8], cross-referencing functional annotations in corresponding records of the effectors in the UniProt/Swiss-Prot database [9], and performing BLAST [10] search against the non-redundant (nr) sequences database.

### Features of T3SEdb

**Figure 1:**
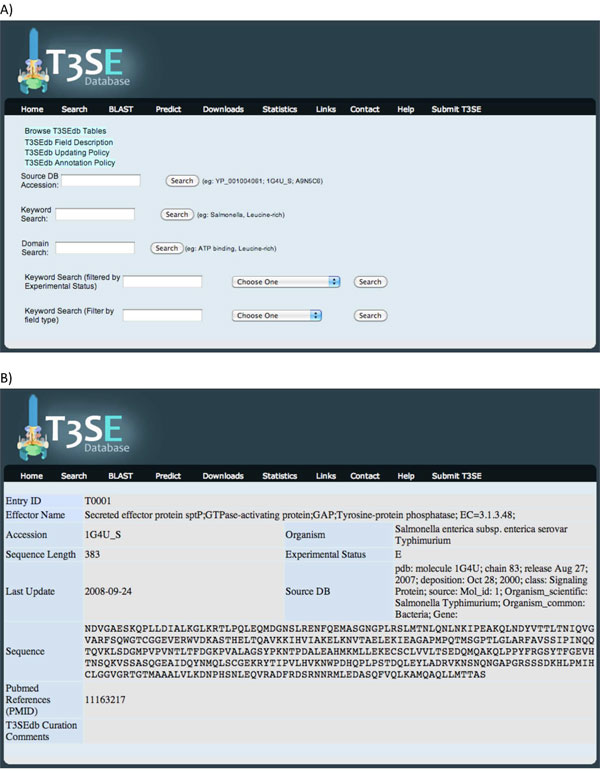
**T3SEdb search function and sample output page**. A) The database can be queried via the NCBI accession number, domain or general keyword search which can also be restricted to the experimental status of the sequences (experimentally validated or hypothetical) or to a specific field in the sequence record. B) Search results display database record with T3SEdb accession number, effector name, hyperlinked NCBI Entrez Protein database accession number, source organism of the effector, sequence length, experimental status, last sequence update, name and accession of the primary/source database that the effector was retrieved from, sequence data, literature references (hyperlinked PubMed IDs) and T3SEdb curation comments (if any).

Users can dynamically browse and string match search the database via the dynamic AJAX/JQuery data request calls to the server. Advanced specific queries are also supported: users can query the database via the NCBI accession number, perform domain or general keyword search, which can also be restricted to the experimental status of the sequences or to a specific field in the sequence record (Figure 1A). Search results are presented in a tabular form, displaying T3SEdb accession number, effector name, hyperlinked NCBI Entrez Protein database accession number, source organism of the effector, sequence length, experimental status, last sequence update, name and accession of the primary/source database that the effector was retrieved from, sequence data, literature references (hyperlinked PubMed IDs) and T3SEdb curation comments (if any) (Figure 1B). Sequence similarity search function against the experimental and hypothetical sequences using the BLAST tool is also provided. Users can batch retrieve sequence data of experimentally confirmed and hypothetical effectors. For curated input to the database by users, a web-interface for submission of new T3SEs is provided with submission and curation review policy indicated http://effectors.bic.nus.edu.sg/T3SEdb/usercurationpolicy.php. A policy on regular update of T3SEdb existing and new records is also defined http://effectors.bic.nus.edu.sg/T3SEdb/updatepolicy.php. Statistics are dynamically updated providing up-to-date general information on the records in the T3SEdb, such as the number of records, the rate of deposition of new effector records into the NCBI Entrez Protein database over the years (1990 to 2010), the list of source species for the effector sequences and the number of experimentally verified and hypothetical sequences classified according to each species.

### Diversity of T3SS effectors

**Figure 2:**
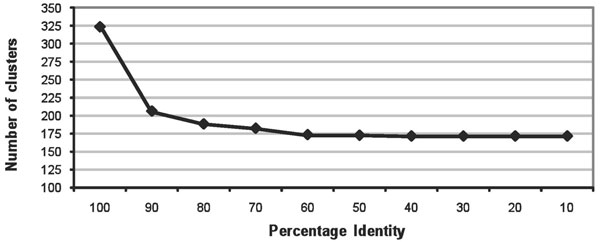
**High sequence diversity of T3SS effectors**. At 100% amino acid sequence identity threshold between the 504 experimentally validated effector sequences, as many as 324 clusters were observed. When the % identity was reduced to 90%, tolerating 10% difference between the sequences, the number of clusters dropped significantly to 206. Allowing more differences between the sequences by reducing the identity threshold (even to as low as 10% identity) did not reduce the number of clusters significantly (171 clusters even at 10% identity). This highlights the high level of amino acid difference between T3SS effectors.

T3SS effector sequences have been reported to be highly diverse [6]. The latest up-to-date collection of experimentally verified effector sequences in T3SEdb enabled assessment of the sequence diversity among them. Clustering of the T3SEs by amino acid difference between the sequences showed that there is a core set of 171 clusters/groups that remained remarkably stable between 10% to 40% identity, as indicated by the clear plateau in Figure 2. This means that there are as many as 171 clusters with amino acid difference of ~60% within clusters and at least ~91% between clusters. This corroborates reports that T3SEs may represent a functionally diverse set of effectors that have a correspondingly wide range of effects on the target host cell [6]. The high level of sequence diversity among T3SS effectors highlights the need for the application of other more conserved functional metadata inherent in the sequences for the construction of models for prediction of novel effectors.

### Prediction of effectors using machine learning algorithms

Machine learning approaches have been used to create tools for prediction of diverse T3SS effectors based on physico-chemical properties, such as hydrophobicity and polarity, in their N-terminal region [11-13], suggesting that these properties are conserved in this region and encode key functional signals to discriminate effectors from non-effectors. Thus the N-terminal region and the inherent physico-chemical properties together with complex machine learning approaches represent attractive avenues for strategies to design and develop T3SS effector prediction models. T3SEdb provides a large number of experimentally known effector sequences with wide species representation for creation of effector predictors which may be useful for scanning of genomes of broad spectrum of bacterial species for discovery of novel T3SEs. We created an effector prediction system to demonstrate the application of the database for such endeavours.

We focused on the 100 amino acids (aa) region of the N-terminal of the experimentally known effectors (positive dataset of 100 sequences from 28 species in the database) and non-effector protein sequences (negative dataset of 100 sequences from 10 species) for the development of the predictor using machine learning methods in the Waikato Environment for Knowledge Analysis (WEKA, version 3.6.2) [14]. Three physico-chemical properties, namely hydrophobicity [15], polarity [16] and β-turns [17] were studied and their scores were ascribed to the overlapping peptides of window size 9 within the 100aa region for both the positive and negative datasets. The application of the physico-chemical property β-turn has not been reported elsewhere and was included because proline residues, which suggest presence of β-turns, are reported to be significantly enriched in effectors of animal pathogens [11], and we also observed enrichment of proline in the N-terminal region of many of the known effectors in the database. Proline residues represented ~5.46% of the amino acids over the 100aa N-terminal region of experimentally validated effectors, which is higher than the UniProt/Swiss-Prot database statistics [18] of ~4.69% for proline.

The performance of several binary classifiers (logistic regression, support vector machines (SVM), naïve Bayes and BayesNet) available in WEKA was evaluated via 10-fold cross-validation. The Naïve Bayes classifier (Table 1) was the best among the other classifiers for the selected feature set of the three physico-chemical properties analysed, with an excellent cross-validation Aroc of ~89%. Testing of the Naïve Bayes model with experimentally validated effectors (positive dataset of 68 sequences from 19 species) and non-effector protein sequences (negative dataset of 68 sequences from 7 species) that were not part of the training data returned an Aroc of ~93%, demonstrating the utility of the model for prediction of effectors. The model has been integrated in T3SEdb as prediction tool http://effectors.bic.nus.edu.sg/T3SEdb/predict.php enabling users to scan sequences of interest against the model for presence of functional signals indicative of a T3SE. The predictive ability of this model is comparable to those developed by others in the field (Aroc of ~86-95%) [11,12].

**Table 1:** Performance measure of binary classifiers in WEKA for prediction of T3SEs.

	Training (10× cross-validation)			Testing			
		
Binary classifier	Aroc	SE	SP	Aroc	SE	SP	PPV
Bayesian Logistic Regression	0.60	0.72	0.49	0.66	0.73	0.60	0.07
Support vector machines (SVM)	0.74	0.97	0.52	0.80	0.95	0.64	0.08
BayesNet	0.86	0.80	0.76	0.91	0.94	0.83	0.15
Naïve Bayes	0.89	0.84	0.82	0.93	0.91	0.83	0.17

SE, SP and PPV refer to sensitivity, specificity and positive predictive value measures, respectively. Aroc is the area under the receiver operator characteristic curve and is commonly used as a measure to assess the quality of a prediction model. The testing Aroc, SE and SP were done with a balanced dataset of 68 effectors and non-effectors that were not part of the training. The PPV was computed using an unbalanced dataset representing the approximate proportion of effectors and non-effectors in a bacterial genome.

The model was also tested for its usefulness in scanning bacterial genomes for novel effectors. However, the test against a dataset approximately proportionate to the ratio of effectors and non-effector protein sequences in a bacterial genome (positive dataset of 49 sequences from 14 species and negative dataset of 929 from 16 species) returned a low positive predictive value (PPV: proportion of true positives over the predicted positives) of ~17%. PPV is a more relevant measure for researchers working in the wet-lab validating predictions because a model with a high PPV would directly result in a significant reduction in effector discovery cost. Though the low PPV for our model is expected given the small proportion of effectors in the bacterial genome, it highlights the challenges facing researchers in the field to develop more sophisticated prediction tools utilizing assemblage of voting of combinations of additional metadata as prediction features to discriminate effectors from non-effectors for practical application in the scanning of bacterial genomes for novel effectors. Examples of such metadata for development of metapredictors may include additional features/criteria such as lower rate of evolution and aggregation propensity, which are characteristics of substrates of chaperones [19] (T3SEs require chaperones for optimal delivery and/or expression [2]), clinical phenotype, host type (plant versus animal), quality of experimental methods used to identify the effectors and BLAST search score of known effectors against bacterial genomes, among others. The T3SEdb represents an excellent platform for inclusion of annotations of such metadata for future developments of sophisticated effector prediction tools applicable for genome scan.

## Conclusions

We have created T3SEdb, the first reported specialised database of T3SS effectors enriched with annotations that facilitated systematic construction of a reliable prediction model for identification of novel effectors. It represents a platform for future developments of sophisticated metapredictors for practical application in the scanning of bacterial genomes for novel effectors.

## Methods

### T3SEdb construction

Bacterial T3SE sequences were collected from the NCBI Entrez Protein database [20] via keyword search. Keyword search was restricted to bacterial sequences and several synonyms of T3SEs (such as Type Three Secretion System effector, Type 3 Secretion System effector, Type III Secretion System effector, TTSS effector, T3SS effector, Type Three Secretion Effector, Type 3 Secretion Effector, Type III Secretion Effector, Type 3 Secreted Effector, Type III Secreted Effector, Type Three Secreted Effector, T3SE, and TTSE) were included in the search to maximise the number of records picked up. The records were downloaded in XML format with the relevant annotation details in the records extracted and tabulated using in house BASH scripts. These were then manually assessed to remove irrelevant records and annotated according to their experimental validation status: E for experimentally confirmed, H for hypothetical or U for unknown.

The clean dataset was then imported into two tables in the MySQL database management system for construction of T3SEdb. One table contains general information about the effector proteins, while the other contains information about the annotations and references. Both tables were integrated together with their NCBI accession number. The Web-based user interface was made with HTML, PHP and jQuery library [21], where HTML and PHP were used for web presentation, PHP to process web forms, and jQuery for AJAX and other JavaScript-based dynamic features. Both the MySQL database and the Web interface are hosted on the cloud server at the National University of Singapore (NUS). The cloud server utilizes a Citrix Xen^® ^Hypervisor running BioSLAX, an open-source Linux Slackware LiveOS distribution developed by the Bioinformatics Centre, NUS, packaged with a comprehensive set of bioinformatics software, Apache, MySQL and PHP (available at [22]).

### Modelling T3SS effector predictor using the annotated data of T3SEdb

Since the prediction was focused on the 100aa N-terminal region of the effector proteins, 8 of the 504 experimentally verified sequences that were shorter than 100 amino acids were removed, resulting in 496 sequences available for analysis. Duplicates were then removed from the remaining sequences to obtain a unique set of 260 sequences. The unique sequences were then clustered using Blastclust [23] at 70% identity threshold, which returned a total of 168 clusters. This was done for better data generalization and to minimize data bias in terms of over-fitting by presence of highly similar sequences and/or by over-representation of data of a particular or a few species. A sequence from each cluster (representing the cluster) was used to form the positive dataset for training of the predictor. A total of 100 non-effector protein sequences, trimmed to their N-terminal 100 amino acids, were used as the negative dataset for the model training. These negative sequences were randomly selected from 10 bacterial species, namely *Citrobacter rodentium*, *Escherichia coli*, *Pseudomonas syringae*, *Pseudomonas tolaasii*, *Salmonella agona*, *Salmonella choleraesuis*, *Salmonella enterica*, *Salmonella typhi*, *Salmonella typhimurium*, and *Yersinia pestis*.

Thereafter, overlapping nonamers of all the sequences from the positive and negative datasets were scored using Protscale [24] for three physico-chemical properties: hydrophobicity using Eisenberg *et al. *scale [15], polarity using the Grantham scale [16] and β-turns using the Levitt scale [17]. This scoring of the nonamers' center position was automated and the original score scale was standardized into a Z-score. The resulting output was 92 features for each individual physico-chemical property, ascribed to each sequence in both the positive and negative datasets.

Following this feature assignment process from Protscale, the features were imported into WEKA for machine learning analysis. WEKA is an integrated package of machine learning algorithms and it provides users with a variety of binary classifiers (algorithms) that can serve as predictors, thus allowing efficient comparison of the different algorithms according to various performance measures after cross-validation. Prior to classification, feature selection using a greedy stepwise algorithm [25] was used to select a reduced feature set of the individual physico-chemical properties. The 92 individual features generated for hydrophobicity, polarity and β-turns were reduced to a total of 63 combined features after feature selection. The performance of a number of classifiers (default parameters setting used) was measured for their ability to classify effectors and non-effectors using the reduced feature set. We performed 10-fold cross-validation on the training dataset (100 effectors and 100 non-effectors) and used the value of the Aroc to compare the performance of the available classifiers. They were then validated using the balanced test dataset of 68 effector and 68 non-effector protein sequences that were not part of the training data for performance measure of Aroc, sensitivity and specificity. The model was also tested against a dataset approximately proportionate to the ratio of effectors (~5% - perhaps an over-estimate) and non-effector proteins in a bacterial genome (~95% - perhaps an under-estimate) to estimate the positive predictive value (PPV).

## Competing interests

The authors declare that they have no competing interests.

## Authors' contributions

DMMT collected, cleaned and annotated the raw data, wrote the scripts and programs used, assisted in the cluster analysis and prediction model building and contributed to the manuscript. KRG set up the database in MySQL, performed the cluster analysis, prediction model building and contributed to the manuscript. AMK participated in the design and coordination of the study, assisted in the prediction model building and drafted the manuscript. TYRO cleaned and annotated the raw data, set up the database architecture in MySQL, designed the web interface and contributed to the manuscript. HMS cleaned and annotated the raw data, performed the cluster analysis, prediction model building and contributed to the manuscript. WWS cleaned and annotated the raw data, performed background search and contributed to the manuscript. MYT cleaned and annotated the raw data, helped in the prediction model building and contributed to the manuscript. FZ cleaned and annotated the raw data and contributed to the manuscript. TWT conceived the study, participated in the design and coordination, wrote scripts and programs for the database browse function and integration of the prediction model into the database, and contributed to the manuscript. All authors read and approved the final manuscript.
